# Immunity after COVID-19 Recovery and Vaccination: Similarities and Differences

**DOI:** 10.3390/vaccines10071068

**Published:** 2022-07-03

**Authors:** Dorota Kamińska, Dominika Dęborska-Materkowska, Katarzyna Kościelska-Kasprzak, Oktawia Mazanowska, Agata Remiorz, Paweł Poznański, Magdalena Durlik, Magdalena Krajewska

**Affiliations:** 1 Department of Nephrology and Transplantation Medicine, Wroclaw Medical University, Borowska 213, 50-556 Wroclaw, Poland; katarzyna.koscielska-kasprzak@umw.edu.pl (K.K.-K.); oktawia.mazanowska@umw.edu.pl (O.M.); agata.remiorz@gmail.com (A.R.); pawel.poznanski@umw.edu.pl (P.P.); magdalena.krajewska@umw.edu.pl (M.K.); 2 Department of Transplantation Medicine, Nephrology and Internal Diseases, Medical University of Warsaw, Nowogrodzka 59, 02-006 Warsaw, Poland; dominika.deborska-materkowska@wum.edu.pl (D.D.-M.); magdalena.durlik@wum.edu.pl (M.D.)

**Keywords:** COVID-19, vaccine, immune response

## Abstract

The coronavirus disease 2019 (COVID-19) pandemic, caused by severe acute respiratory syndrome coronavirus 2 (SARS-CoV-2), is associated with a robust immune response. The development of systemic inflammation leads to a hyperinflammatory state due to cytokine release syndrome during severe COVID-19. The emergence of many new SARS-CoV-2 variants across the world deteriorates the protective antiviral immunity induced after infection or vaccination. The innate immune response to SARS-CoV-2 is crucial for determining the fate of COVID-19 symptomatology. T cell-mediated immunity is the main factor of the antiviral immune response; moreover, SARS-CoV-2 infection initiates a rapid B-cell response. In this paper, we present the current state of knowledge on immunity after COVID-19 infection and vaccination. We discuss the mechanisms of immune response to various types of vaccines (nucleoside-modified, adenovirus-vectored, inactivated virus vaccines and recombinant protein adjuvanted formulations). This includes specific aspects of vaccination in selected patient populations with altered immune activity (the elderly, children, pregnant women, solid organ transplant recipients, patients with systemic rheumatic diseases or malignancies). We also present diagnostic and research tools available to study the anti-SARS-CoV-2 cellular and humoral immune responses.

## 1. Introduction

The severe acute respiratory syndrome coronavirus 2 (SARS-CoV-2) is a positive-sense single-stranded (ss) RNA virus that belongs to the betacoronavirus 2B lineage of the coronavirus family [1]. As in the case of all coronaviruses, SARS-CoV-2′s entry into host cells is mediated by spike glycoprotein (S protein). The S protein of SARS-CoV-2 contains a receptor-binding domain (RBD) that specifically recognizes the angiotensin-converting enzyme-2 (ACE2) receptors. RBD is a critical target for antiviral compounds and antibodies; however, nucleocapsid protein (N) and other structural or non-structural SARS-CoV-2 proteins can elicit the host immune response as well [2,3].

SARS-CoV-2 infects various cells that express ACE2 receptors (oropharyngeal mucosal and endothelial cells, pneumocytes, gastrointestinal tract and kidneys) [4,5]. The binding of the virus to the endothelial cells leads to endothelium dysfunction followed by microvascular thrombotic and inflammatory processes, which are responsible for the clinical picture of COVID-19 [6]. 

Numerous SARS-CoV-2 variants of concern have been identified over the two years of the pandemic, including B.1.1.7 (Alpha, 20I/501Y.V1 first identified in United Kingdom [7]), B.1.351 (Beta, 20H/501Y.V2, first identified in South Africa [8]), P.1 (Gamma, first identified in Brazil [9]), B.1.617.2 (Delta, first identified in India [10]) and B.1.1.529 (Omicron, first identified in South Africa [11]). Some additional variants of interest have also been listed by the World Health Organization.

### Literature Search

**Figure 1:**
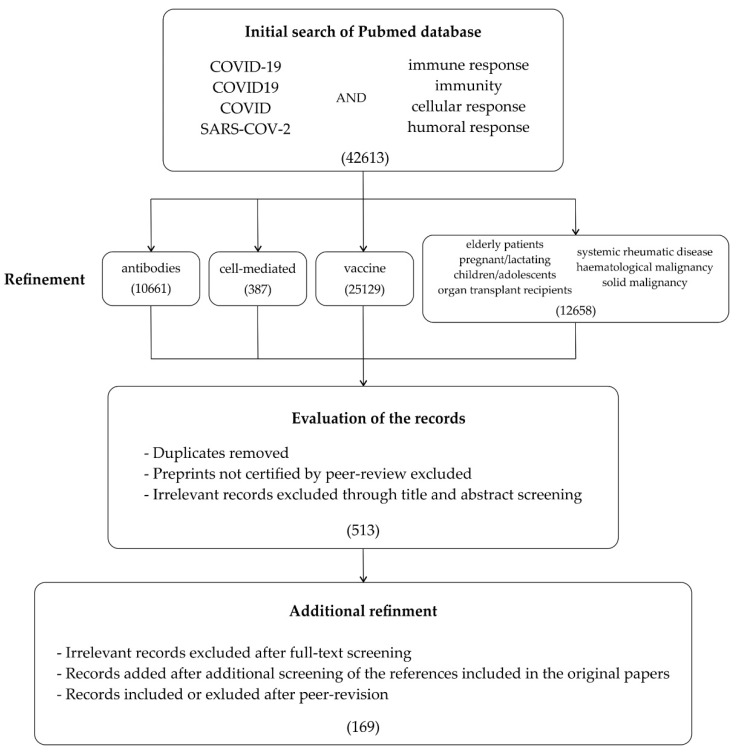
The summary of the literature search on the immunology of SARS-CoV-2 infection and vaccines.

The authors performed a review of the literature on the immunology of SARS-CoV-2 infection and vaccines on PubMed based on the following keywords: “immune response”, “immunity”, “cellular response”, “humoral response” and “vaccine”, combined with at least one of the following terms: “COVID-19”, “COVID”, “SARS-CoV-2”, “COVID19” between 1 January 2020 and 18 June 2022. Only peer-reviewed, English-language essential research papers were included. An additional screening of the references included in the original papers was performed to obtain supplementary studies. The manuscript selection process is summarized in Figure 1.

## 2. Diagnostic and Research Tools to Study the Anti-SARS-CoV-2 Immune Response

There is a wide range of available diagnostic and research assays that provide a measure of anti-SARS-CoV-2 specific immune responses. The most accessible are the humoral immunity assays including lateral flow immunoassays (LFIA) [12,13], chemiluminescence immunoassays (CLIA), as well as enzyme-linked immunosorbent assays (ELISA). Out of the four structural proteins of SARS-CoV-2 [1], most currently available serology assays are designed to assess antibodies against S and N proteins.

LFIA is a broadly distributed point-of-care test—the simplest qualitative test that allows even a non-professional to interpret its results. Some of the cassette assays include Accu-Tell COVID-19 IgG/IgM Antibody Test (AccuBiotech Co., Ltd., Beijing, China), COVID-19 Spring IgM/IgG Rapid Test Cassette (Spring Healthcare Services AG, Zug, Switzerland) or COVID-19 IgG/IgM Rapid Test Cassette (SureScreen Diagnostics Co., Ltd., Derby, Great Britain) [14], which have proven to be suitable for the rapid identification of antibody responses, but have the lowest significance in scientific research. 

ELISA is a plate-based qualitative or semi qualitative immune assay that uses an antigen-coated plate for enzyme-driven detection of the plate-bound antigen-specific serum or plasma antibodies, which is adaptable to automation. There are many commercially available anti-SARS-CoV-2 ELISAs, including Euroimmun Anti-SARS-CoV-2 ELISA IgG, IgA or IgM assays, which have been introduced into scientific and clinical practice [13,15,16,17,18,19,20].

CLIA is the highest-throughput qualitative antibody detection assay also available for anti-SARS-CoV-2 serology testing. Examples of CLIAs available on the market include LIAISON™ SARS-CoV-2 S1/S2 IgG (DiaSorin, Saluggia, Italy); ARCHITECT™ SARS-CoV-2 IgG (Abbott, Abbott Park, IL, USA); VITROS™ Anti-SARS-CoV-2 Total (Ortho-Clinical Diagnostics, Raritan, NJ, USA); SARS-CoV-2 Total Assay (Siemens, Malvern, PA, USA); Elecsys™ Anti-SARS-CoV-2 (Roche, Rotkreuz, Switzerland). Among the key advantages of CLIAs are also their wide dynamic range, high signal intensity, high specificity and rapid acquisition of the analytical signal [21].

The plaque reduction neutralization test (PRNT) is the gold standard for the quantification of neutralizing antibodies. In a PRNT, the neutralizing activity of a serum sample is assayed by incubating the sample with a viral suspension, which is followed by application of the mixture onto a cell culture monolayer, and after a few incubation days, the enumeration of plaques, i.e., regions of infected cells, is performed. The concentration of serum needed to reduce the number of plaques by 50% compared to the serum-free virus suspension gives the measure of how much antibody is present or how effective it is. 

The pseudo-neutralizing antibody assay, also known as the surrogate virus neutralization test (sVNT), is an alternative to PRNT that detects neutralizing antibodies without the need for any live virus or cells, and as such, is accessible to any BSL2 laboratory [22]. In sVNT, the real SARS-CoV-2—ACE2 receptor binding is mimicked in vitro via a protein–protein interaction using purified recombinant human ACE2 and the RBD of the SARS-CoV-2 S protein. This interaction can be blocked by neutralizing antibodies present in the test serum and their level is assessed using comparative ELISA. sVNTs are available commercially and include SARS-CoV-2 NeutraLISA (Euroimmun, Lubeck, Germany), TECO SARS-CoV-2 Neutralization Antibody Assay from TECOmedical (Sissach, Switzerland), and cPass™ SARS-CoV-2 Neutralization Antibody Detection KIT (GenScript, Piscataway Township, USA).

The SARS-CoV-2-specific T cell response can be characterized with the use of ELISpot, flow cytometry, and interferon-gamma release assays (IGRA). 

IGRA is a whole-blood assay based on the ELISA assessment of the level of interferon-gamma released upon stimulation with target antigens—either recombinant viral proteins [23] or synthetic peptide pools [24,25]. Numerous IGRAs are commercially available, including QuantiFERON SARS-CoV-2 assay (Qiagen, Düsseldorf, Germany) and Quan-T-cell SARS-CoV-2 (Euroimmun, Lubeck, Germany).

While a laboratory can introduce IGRA into its portfolio quite easily, the other methods are more laborious and require specialized instrumentation. The enzyme-linked immunosorbent spot (ELISPOT) assay is a highly sensitive and specific immune monitoring tool that quantifies cytokine-producing T lymphocytes that are reactive to a given antigen. In the case of SARS-CoV-2-directed immunity, peripheral blood mononuclear cells (PBMCs) or enriched populations are co-cultured with synthetic SARS-CoV-2 peptides on a membrane-bottomed plate [26,27]. The induced release of the cytokine (interferon-gamma or interleukin-2 are typically used) and its visualization on a membrane leads to the formation of the spots. The level of induced immune response is described as spot-forming units, which are automatically counted for a given number of cultured cells. 

Flow cytometry enables the detailed analysis of a panel of cytokine-producing cells after exposure to the virus antigens (peptide pools) [28,29]; however, it is more significant in research than in diagnostic use. 

Rapid development of numerous methods for the serological assessment of SARS-CoV-2 specific immunity over the course of COVID-19 pandemic posed a need to establish assay and reporting units standardization to facilitate the cross-comparison of results across studies, as well as for streamlined meta-analyses [30,31]. 

The standardization was also crucial for the establishment of a universal measure of protection to broaden the clinical utility of serological testing for patient care, vaccine trials and the optimization of vaccination regimes for protection of general and special populations. In December 2020, the National Institute for Biological Standards Control released the First WHO International Standard for anti-SARS-CoV-2 immunoglobulin (human, NIBSC code: 20/136) [32]. As a result, the reporting of semiquantitative or quantitative results of serology testing in binding antibody units (BAU) per milliliter traceable to the WHO standard was introduced in the laboratory practice. When interpreting the results presented in non-standardized units, the assay-specific cutoffs and ranges should be considered. 

## 3. Immune Response to SARS-CoV-2 Infection

### 3.1. Innate Immune Response to SARS-CoV-2 Infection

The innate immune response to SARS-CoV-2 is crucial for determining the fate of COVID-19 symptomatology [33,34]. Innate immune system function depends on several types of cells, including dendritic cells, monocytes, macrophages, neutrophils, eosinophils, mast cells and natural killer cells (NK). They are all able to recognize host-derived molecules released from damaged or dying cells, which are known as pathogen-associated molecular patterns (PAMPs) and damage-associated molecular patterns (DAMPs) [35]. 

Pattern recognition receptors (PRRs) include toll-like receptors (TLRs), retinoic acid-inducible gene I (RIG-I)-like receptors (RLRs) and nucleotide-binding oligomerization domain (NOD)-like receptors (NLRs). PRRs are engaged to detect specific viral components, such as viral nucleic acids, and to induce type I interferons (IFNs). Type I IFNs are the principal cytokines involved in the antiviral response. After the recognition phase, an effector phase of the innate immune response initiates and engages several soluble mediators, e.g., cytokines, chemokines and the complement system, which leads to the elimination of infected cells [33,36,37]. Under optimal circumstances, abrupt IFN-I production by infected cells occurs immediately after infection and limits viral replication within a few hours or days [38].

SARS-CoV-2 has developed several mechanisms to overcome the IFN pathway of the immune response [39] and to stimulate the hyperproduction of proinflammatory cytokines and chemokines via NF-κB activation [40]. An immune phenotype consisting of a highly impaired IFN type I response (no IFN-β and low IFN-α production and activity) together with persistent blood viral load and an exacerbated inflammatory response was observed in severe and critical COVID-19 patients [41]. In the first phase after SARS-CoV-2 infection, the virus can impair the capacity of infected cells to produce interferons [42]. In the second phase, the immune system regains its ability to defeat the virus effectively, but an overreaction overcomes the naturally existing limiting mechanisms.

In summary, the innate immunity response against SARS-CoV-2 infection strongly depends on PAMPs/DAMPs–PPRs interactions and IFN-I-dependent responses. An impaired IFN-I response leads to the hyperproduction of proinflammatory cytokine-storm-related clinical symptoms. 

### 3.2. Cytokine Storm in COVID-19

Overall, an exuberant innate immunoinflammatory response is a hallmark of severe COVID-19 with the cytokine storm, hyperinflammation, multiorgan failure, and acute respiratory distress syndrome [42]. Increased generation of pro-inflammatory markers in severe COVID-19 is a basis for cytokine storm and an enhanced mortality risk [43]. Numerous studies stated that the severity of COVID-19 symptoms are correlated with elevated blood levels of proinflammatory cytokines and chemokines, e.g., IL-1b, IL-2, sIL-2RA, IL-6, IL-7, IL-17, IL-18, TNF-α, monocyte chemoattractant protein 1 and 3 (MCP-1 and 3), cytosolic carboxypeptidases 2 and 3 (CCL2 and 3), granulocyte colony-stimulating factor (G-CSF), interferon gamma-induced protein 10 (IP10), macrophage colony-stimulating factor (M-CSF) and microprotein 1a (MIP-1a) [44,45,46]. Infected pulmonary epithelia release IL-1b, IL-6 and IFN-I/III, which contribute to macrophage activation and the recruitment of monocytes, granulocytes and lymphocytes from the blood. Sustained IL-6 and TNF-α release by influent monocytes causes hyperinflammation cascades and cytokine release syndrome. The deviant immune response leads to a secondary hemophagocytic lymphohistiocytosis-like reaction followed by microthrombosis, neutrophilic NETosis and the recruitment of effector immune cells [47]. 

Clinically, cytokine storm is characterized by high fever, fatigue, headache, dyspnea, respiratory distress, cardiomyopathy, liver damage, acute kidney injury, capillary leak syndrome, as well as encephalopathy, and thrombotic complications evolving in disseminated intravasal coagulopathy [48]. Routine laboratory markers reveal lymphopenia (especially T cell depletion), high serum ferritin, D-dimers, C-reactive protein (CRP) and cytokines, including IL-1b, IL-6, and TNF-α [49,50]. 

In summary, an inadequate balance between the production of pro-inflammatory and anti-inflammatory cytokines leads to the dysfunction of the complex mechanism responsible for the extinction of the immune reaction. Cytokine storm followed by T cell depletion leads to an exaggerated and persistent inflammatory state, which may progress to multiorgan failure and death. 

### 3.3. Cell-Mediated Response to SARS-CoV-2 Infection

As innate lymphoid effector cells, natural killer (NK) cells rapidly respond to SARS-CoV-2 infection through cytokine production and the direct lysis of infected cells. High levels of IL-6 correlate with a lower number of NK cells in COVID-19 patients [51], whereas a decrease in NK cell frequency correlates with COVID-19 disease severity [52,53]. NK cells are activated by immunoglobulin G1 (IgG1) and IgG3 antibodies through Fc receptors. This suggests that antibodies targeting IL-6 and TNF-signalling may restore NK cell functions in COVID-19 patients [47].

The anti-viral immune response depends on T cells. CD8+ T cells destroy infected cells, whereas CD4+ T cells stimulate B cells to produce antibodies. Lymphopenia with a deficit of CD4+ T cells and a higher number of CD8+ T cells correlate with mortality in severe SARS-CoV-2 infection [54]. During a persistent viral infection, T cells enter a dysfunctional state known as T cell exhaustion. A COVID-19-related impairment in CD4+ T cells promotes the excessive activation and possibly subsequent exhaustion of CD8+ T cells [51,55]. 

A lower number of T cells at COVID-19 onset was reported to be a marker of progression to severe disease [56]. A decreased level of T helper cells, suppressor cells, T regulatory cells, as well as NK cells, is correlated with COVID-19 severity. The IL-2/INF-γ ratio was the strongest indicator of a critical course of COVID-19 and was associated with fatal outcomes [57]. After recovery from SARS-CoV-2, sustained low levels of CD4+ T cells, B cells and granulocytes were detected. Decreased levels of CD8+ regulatory T cells, and increased levels of CD56+CD16− NK T cells with the promotion of a Th17-type phenotype, resulted in a persistent proinflammatory response [58]. Virus-specific CD4+ and CD8+ T cells are polyfunctional and survive with an estimated half-life of 200 days. CD4+ T cell responses target several SARS-CoV-2 proteins, whereas the CD8+ T cell responses preferentially target the nucleoprotein [29]. The level of CD8+ T cell response decreases to almost half when compared to CD4+ after 6 months of infection. Anti-SARS-CoV-2 memory response is predominated by CD4+ T cells [59]. 

In summary, the cellular anti-SARS-CoV-2 response starts from NK cells through cytokine production (mainly IL-6) and the direct lysis of infected cells. Subsequently CD8+ T cells destroy infected cells, whereas CD4+ T cells stimulate B cells to produce antibodies. However persistant infection lead to further T cell exhaustion with lymphopenia with a high CD8+/CD4+ ratio as a major hallmark of progression to severe COVID-19.

### 3.4. Humoral Response to SARS-CoV-2 Infection

SARS-CoV-2 infection initiates a rapid B cell response, with virus-specific IgM, IgG, and IgA antibody production and a neutralizing capacity during the first 19 days after symptom onset [60]. The S protein receptor-binding domain (RBD) is highly immunogenic, and anti-RBD antibodies block virus interactions with the entry receptor, namely ACE2 [61]. Analysis of epitope distribution along the spike gene sequence demonstrated that most IgM- and IgG-reactive peptides were clustered into similar genomic regions. The humoral immune system recognized few conserved S protein epitopes in most COVID-19 patients. Seven S peptides were generally recognized by IgG antibodies after SARS-CoV-2 infection [56]. Anti-SARS-CoV-2 antibodies were present in 85% of patients 4 weeks after the onset of COVID-19. The levels of specific IgM and IgA antibodies decreased after 1 month while anti-SARS-CoV-2 IgG antibodies were still present, although at a significantly lower level, in 80% of patients at 6–8 months after symptom onset [27].

Measures of IgG and IgM antibodies did not predict disease severity [62], however, the peak levels of neutralizing antibodies were positively associated with the severity of COVID-19 [63]. Higher titers of virus-specific antibodies were detected in patients with severe COVID-19, including deceased patients, when compared to asymptomatic patients [64]. A recent small study from China reported that SARS-CoV-2 antibody titers were higher in patients who were aged >60 years and with severe symptoms [65]. 

However the highly elevated IgG levels against S glycoprotein positively correlated with biomarkers of immune activation and negatively correlated with pulmonary function and the extent of pulmonary CT abnormalities in COVID-19-convalescents [66]. Higher peak antibody titers and disease severity were associated with an increased durability of detectable anti-spike IgG antibodies titers detectable one year after hospitalization for COVID-19 [67]. Early non-neutralizing IgG responses may play a key role in complement overactivation in severe COVID-19. Complement activation in the classical pathway in COVID-19 patients is associated with a worse disease outcome. Plasma levels of C1q were increased in severe COVID-19 patients and correlated with higher IgG titers [68].

Neutralizing antibody titers were generally maintained for a period of 3–6 months and gradually decreased after 5 to 8 weeks, but were still detectable up to 8–9 months in most recovered patients [69]. The lack of neutralizing capacity correlated with an increased risk of a fatal outcome [70]. Another study showed that 93 to 97% of patients were positive for anti-SARS-CoV-2 RBD IgG 1 year after infection [68,71]. A recent study showed that the majority of the COVID-19 convalescents maintained detectable, with anti-RBD IgG and neutralizing activity at 400–480 days after COVID-19 onset [72]. In survivals to critical illness, the titers of IgG and neutralizing antibodies remained high at 12 months, but the levels declined during this period by over 50% [73]. Anti-SARS-CoV-2 spike- and internal protein-specific T cellular responses were reported in patients with persistent antibodies 6 months after infection. IL-2 and IFN-γ-secreting T cell responses, as well as levels of specific IgG and neutralizing antibodies, were correlated with disease severity [74]. Current studies report a decline in neutralizing antibody responses in convalescent patients and documented cases of SARS-CoV-2 reinfection [75]. SARS-CoV-2 symptomatic reinfection was also reported 45 and 141 days after initial infection despite the presence of IgG antibodies [76].

Memory B cells can immediately respond to reinfection by generating new high-affinity plasma cells, which is essential for long-lasting immunity. The durability of protective humoral immunity after SARS-CoV-2 infection is largely dependent on the persistence of antigen-specific memory B cells and long-lived plasma cells secreting high-affinity neutralizing antibodies that reside in the bone marrow. The number of spike-specific memory B cells increased with time after symptom onset, whereas SARS-CoV-2-specific CD4+ T cells and CD8+ T cells declined, with a half-life of 3–5 months [77]. Baseline antigen-specific CD4+ T cell response is a predictor of the maintenance of antibody neutralization breadth and RBD-specific memory B cell levels at 12 months post-infection [78]. In mild COVID-19 convalescents, the peak memory B cell response was detected at 3 months after symptom onset and persisted up to 7 months after infection. Significant memory T cell levels were detected 1 month after disease onset and persisted even when specific humoral immunity declined [79]. The level of immune responses at 1 year post-COVID-19, mainly the phagocytic capacity and memory B cell responses, depended on the severity of the prior COVID-19 [80].

It was also reported that anti-Spike(S) protein IgG antibodies are present in oral mucosal fluids in individuals recovered from COVID-19. Anti-SARS-CoV-2 antibody responses were detected in serum and saliva, with peak IgG levels reached by 16–30 days after the onset of COVID-19 symptoms. Long-term observation revealed that anti-SARS-CoV-2 IgA and IgM antibodies rapidly decayed, while IgG antibodies remained relatively stable up to 3 months post infection. Mean titers of IgG, IgM and, to a lesser extent, IgA responses to spike and RBD in the serum positively correlated with matched saliva samples [81]. The average IgG titers noticed by Chellamuthu et al. remained relatively stable over a period of nearly one year post-infection. Participants of the study who were vaccinated during the observation period presented mean IgG antibody concentration at the first time point post vaccination higher than the maximum observed for post-infectious IgG concentration [82]. Another study of Mades et. al. showed that 100% participants developed detectable oral mucosal SARS-CoV-2 IgG antibodies by 15 days after the first vaccination dose with the mRNA vaccine [83]. 

In summary SARS-CoV-2 infection initiates a rapid B cell response, with virus-specific IgM, IgG, and IgA antibody production and neutralizing capacity against the RBD domain of the S protein and N protein. Neutralizing antibody titers are generally stable for a period of 3–6 months and then gradually decline. The robustness of humoral protection after SARS-CoV-2 infection is largely dependent on the persistence of antigen-specific memory B cells and long-lived plasma cells.

### 3.5. Medications Influencing the Severity of SARS-CoV-2 Infection

There is no consesus about drugs influencing the course of SARS-CoV-2 infection. One of particularly vulnerable populations with a high risk of severe infection and high infection-related mortality rate are immunocompromised patients [84]. Solid organ transplant recipients, treated with immunosuppressive drugs, were declared a group with a high risk for severe COVID-19 [85]; on the other hand, it was postulated that the hyperinflammatory state with cytokine release syndrome during severe COVID-19 could be alleviated by immunosuppressive therapy in transplant recipients. Santeusanio et al. reported that immunosuppression intensity following a COVID-19 diagnosis was not associated with mortality [86].

Synthetic glucocorticoids are widely used in the therapy of autoimmune, endocrine, hematological, oncological and inflammatory diseases. In the case of severe infection, allergy, shock etc., the supplementation of glucocorticoids is critical to protect the functions of vital organs. After the initial uncertainty during the onset of COVID-19 pandemic, glucocorticosteroids, mainy dexamethasone, are a standard therapy in severe disease course [87]. However, due to their immunosuppressive potential and potential harms, high doses of corticosteroids should be avoided early in the disease course, when patients do not require oxygen support [88].

Anti-CD20 agents are widely used in rheumatoid, hematological, neurological and autoimmune diseases. Recent analysis of studies on patients with multiple sclerosis treated with rituximab revealed that they are at higher risk of severe COVID-19 outcomes when compared to patients under other treatments [89]. Additionally, ocrelizumab therapy was shown to be connected with prolonged COVID-19 [90]. Recent study on immune-modifying therapies in immune-mediated inflammatory diseases showed that there was no evidence of increased COVID-19-related death in adults treated with tumour necrosis factor inhibitors, interleukin (IL)-12/IL-23 inhibitors, IL-17 inhibitors, IL-6 inhibitors, or Janus kinase inhibitors when compared with those on standard systemic therapy [91].

## 4. Immune Response to SARS-CoV-2 Vaccination

The worldwide vaccination effort has become a major weapon in overcoming the COVID-19 pandemic and reducing the number of its victims. Currently, there is a range of vaccines for which the World Health Organization (WHO) [92] has issued an emergency-use listing (EUL) (Table 1). The portfolio includes the mRNA (nucleoside modified), adenovirus-vectored, recombinant protein and inactivated formulations. Different types of vaccines induce various types of immune responses, resulting in varying efficiencies. Except for the inactivated ones, all EUL vaccines cause the expression of the full-length S protein of SARS-CoV-2 on the membrane of the cell that takes up the mRNA-containing nanoparticles, or the infection of the cell with the adenovirus vector.

### 4.1. Nucleoside-Modified Vaccines

Only two of the WHO EUL vaccines (BNT162b2—Comirnaty, Pfizer/BioNTech, Mainz, Germany; mRNA-1273—Spikevax, Moderna, Madrid, Spain) were approved by the FDA (Food and Drug Administration). All five EMA (European Medicines Agency) authorized vaccines are still conditionally approved. (Table 1). 

The active component of new RNA-based EUL vaccines is an RNA strand modified by incorporating 1-methyl-pseudouridine, which dampens innate immune sensing and increases mRNA translation in vivo. The vaccine RNA is encapsulated in lipid nanoparticles for more-efficient delivery into cells after intramuscular injection. SARS-CoV-2 vaccines are designed to deliver an antigenic sequence of the spike protein with some modifications that were introduced to stabilize their protein products [93,94]. The take-up of the vaccine by a cell results in an expression of the SARS-CoV-2 antigen that can be processed for presentation via class I and II MHC from the transfected cells and professional antigen-presenting cells, respectively [95,96]. As a result, a Th1-cell-skewed immune response can be generated with antigen-specific CD4+ T helper and CD8+ cytotoxic T cells, along with neutralizing antibody response from B cells. The vaccination-induced activation of the innate immune cells is also required to activate the lymphocytes to obtain both B and T cell responses. In the case of the mRNA vaccines, the adjuvant activity is related either to mRNA molecules (BNT162b2) or to the lipid structure of nanoparticles (mRNA-1273) [97,98]. As no viral vector is used to deliver the antigenic sequence, the risk of pre-existing immunity, which may diminish efficacy, is eliminated.

In the initial study, BNT162b2 was shown to induce a broad immune response with SARS-CoV-2 S-specific neutralizing antibodies and poly-specific CD4+ and CD8+ T cells within the 9-week observation period after the two-dose vaccination. The study participants were vaccinated with BNT162b2 mounted de novo S-specific CD4+ T cell responses, and nearly 90% of them also with de novo CD8+ T cell responses. The robust expression of IFNγ and IL-2 and low levels of IL-4 in BNT162b2-induced CD4+ T cells indicated a T helper 1 cell profile [99].

The authors of another trial with two 30µg doses of the BNT162b2 vaccine reported a robust initial increase in S-RBD IgG levels (with median IgG levels of 1246 AU/mL 21 days after the first dose), which increased significantly, reaching the maximum level at 7 days after the second dose (24,534 AU/mL) [100]. At six months post-vaccination, on average, the S-RBD IgG levels decreased to 7% of their peak level. The post-infection IgG levels (median 1532 AU/mL) were similar to those exhibited by vaccinated persons 21 days after the first dose and by fully vaccinated persons six months after the second dose; nonetheless, they were significantly lower than in double-vaccinated individuals tested 1–12 weeks after the second dose.

87% of vaccinated individuals developed either SARS-CoV-2-specific memory CD4+ or CD8+ T cell responses 12 weeks after the second dose. The frequency of S-specific CD4+ T cells was higher than that of corresponding CD8+ T cells. There was no significant correlation between S-RGD IgG and T cell responses. The inhibition of the spike–ACE2 interaction was observed in most of the serum samples 12 weeks after the 2nd dose. The inhibition was weaker with the Beta and Gamma variants of the virus and only slightly weaker with Delta and Kappa when compared to the wild type of SARS-CoV-2 [100]. 

At the peak of a response to the second mRNA-1273 vaccine dose, all individuals had responses to all SARS-CoV-2 variants (Alpha, Beta, Gamma, and Delta) on binding, neutralizing, and ACE2-competing antibodies. Nevertheless, across all the assays, Beta had the lowest antibody recognition. Neutralizing antibodies to S-RBD activity against all variants peaked two weeks after the second dose with a moderate decline over time through day 209. Individuals who demonstrate waning immune responses over time are likely to have memory B cells capable of delivering an anamnestic response to those variants in the event of exposure to the virus, or potentially with an additional dose of vaccine [101].

As a result of a global, randomized, placebo-controlled, Phase 1–2–3 pivotal trial, in which two 30-μg doses of mRNA vaccine were administered 21 days apart, the authors suggested that a third dose provided approximately 8 months after the second dose could prolong protection and further increase its scope. After 1 month following the third dose, the mean neutralization antibody titers against wild-type virus increased to more than five to seven times as compared to titers observed 1 month after the second dose. After the third dose, neutralization antibody titers against the Beta variant increased more than against the wild-type virus (15 to 20 times) when compared to the titers after the second dose [102]. 

In another study, the authors reported reduced neutralizing activity against the Omicron variant (only 21% of patients) as compared with the Beta variant and suggested a need for a booster dose to maintain neutralizing activity against the Omicron variant. At 2 to 4 weeks after a primary two-doses series of mRNA vaccinations, they observed a 30-fold reduction in neutralizing activity against Omicron. Six months after the initial two-vaccine doses, sera from naïve vaccinated subjects show no neutralizing activity against the Omicron. On the contrary, most COVID-19-recovered patients retained neutralizing antibody responses 6 months after receiving the primary series of vaccinations (showing a 22-fold reduction in neutralization activity). One to four weeks after the third vaccine dose, over 90% of subjects showed neutralizing activity against the Omicron variant, with a 2.4-fold and 14-fold reduction in neutralizing activity as compared to the wild type; however, more than 90% of subjects retained neutralizing activity against the Omicron variant [103].

### 4.2. Adenovirus-Vectored Vaccines

Yet another newly approved technology, also leading to Th1-cell-skewed response, is the adenovirus-vectored SARS-CoV-2 vaccines that mimic the features of natural viral infection (ChAdOx1-S or AZD1222—Vaxzevria, AstraZeneca, London, Great Britain; Covishield, Serum Institute of India, Pvt, Ltd., Pune, India; Ad26.COV2-S or JNJ-78436735—Jcovden, Janssen Pharmaceutica, Beerse, Belgium). As a result of adenovirus-vector infection, an infected cell expresses the vector-encoded S protein of SARS-CoV-2 on the cell membrane. The Janssen vaccine is based on human adenovirus serotype 26 (Ad26), which uses CD46 to enter the cells. In this case, there is a possibility for the successful delivery of the antigen-encoding DNA to the nucleus of the target cell, including DCs. Other adenovirus-vectored vaccines use chimpanzee adenovirus-derived ChAdOx1, which infects the target cells via the coxsackie adenovirus receptor, which is expressed on a limited number of cell types [104]. The source of the S protein antigen generated by ChAdOx1 based vaccination appears to be dead cell fragments likely originating from infected endothelial cells [105]. The possible scenario following a ChAdOx1-based vaccination is an immune response to ChAdOx1, followed by a ChAdOx1-specific attack on vector-infected endothelial cells and the subsequent uptake of the S protein-containing dead endothelial cell fragments for antigen presentation by APCs, and finally—the S protein-specific B cell and CD4+/CD8+ T cell immune responses.

In the phase I study, a single immunization with Ad26.COV2.S induced rapid binding and neutralization antibody responses [106]. In 90% of vaccine recipients, binding antibodies emerged rapidly by day 8 after initial immunization and neutralizing antibodies were observed in 25% of individuals at the same time. By day 15, after a single vaccine dose, S-specific and RBD-specific binding antibodies were observed in 100% and neutralizing antibodies in 85% of patients. Antibodies against the full-length S protein were noticed in 65% of vaccine recipients 8 days after vaccination. By day 57, 100% of vaccine recipients showed neutralizing antibodies, as well as S- and RBD-specific binding antibodies. S-specific cellular immune responses assessed by IFN-γ ELISpot were observed in 65% of patients by day 15 and increased to 84% by day 71. Cellular responses correlated with antibody titers on day 29 (S-specific binding, RBD-specific binding and neutralizing antibody). The booster vaccine dose given on day 57 resulted in a nearly 2.56-fold increase in binding antibody titers and an almost 4.62-fold increase in neutralizing antibody titers (measured on day 71).

After two doses of the ChAdOx1 nCoV-19 vaccine (28 days apart), median anti-spike SARS-CoV-2 IgG responses 28 days after the booster dose were 20,713 arbitrary units [AU]/mL. Neutralizing antibody median titers as assessed by live SARS-CoV-2 microneutralization assays peaked by day 42. There was no difference in antibody response between older and younger participants. Two weeks after the booster dose, >99% of participants had neutralizing antibody responses. T cell responses, assessed using an ex-vivo IFN-γ ELISpot assay, peaked at day 14 after a single standard dose of vaccine (a median of 1187 spot-forming cells, SFCs, per million peripheral blood mononuclear cells) and did not increase significantly after the booster dose [107].

A study by Halperin et al. [108] on a novel recombinant coronavirus vaccine (adenovirus type 5 vector) showed that a single dose of Ad5-nCoV vaccine elicited a substantive anti-spike antibody response 28 days after vaccination with a mean antibody titer increase of 32-fold from prevaccination to postvaccination and seroconversion shown in 91.5% of recipients. The neutralizing antibody response showed a mean fold increase of 11.4, with seroconversion of neutralizing antibodies shown in 75.9% of individuals. 

Since mutations can decrease the neutralization potency of an antibody, the efficacy of the heterologous prime-boost vaccination schedule was determined. It was assumed that heterologous prime-boost regimens can induce equal or even stronger immune responses against the novel viral variants when compared to the homologous prime-boost regimens. Heterologous ChAdOx1 nCoV-19/BNT162b2 vaccination led to a significant 11.5-fold increase for anti-S IgG, compared to a 2.9-fold increase after homologous ChAd vaccination [109].

### 4.3. Inactivated-Virus Vaccines

The difference between inactivated whole-virion formulations (CoronaVac, Sinovac Life Sciences; BBV152—Covaxin, Bharat Biotech; BBIBP-CorV—COVID-19 Vaccine BIBP aka Covilo, SinoPharm) and mRNA or adenoviral-vectored vaccines is that the viral antigens (S, M and N structural proteins) from inactivated virions can be directly presented to DCs after their uptake through the lysosomal pathway. Inactivated-virus vaccines are similar to the live virus infection; however, they offer a limited duration of the presented antigens and lack dead infected cell fragments for MHC I presentation of the viral antigens. The results of phase I/II clinical trials of CoronaVac have shown low IFNγ-expressing SARS-CoV-2-specific T cell responses, suggesting a Th2-skewed T cell response to vaccination [110].

The result of randomized, double-blind, placebo-controlled, multicenter, phase I, II, and III clinical trials showed the inactivated-virus vaccines to be safe and well-tolerated at doses of 1.5, 3, and 6 μg when given in two doses (days 0 and 28) in people older than 60 years. Seroconversion 28 days after the second dose was observed in 100.0% of individuals belonging to the 3 μg group and 95.7% of individuals in the 6 μg group. In phase II, seroconversion was seen in 90.7% of individuals in the 1.5 μg group, as well as in 98% of those in the 3 μg group, and 99% of those in the 6 μg group. The neutralizing antibody responses observed in individuals who received the 3 or 6 μg dose were higher than in those who received the 1.5 μg dose [111].

A study on BBV152, a whole-virion inactivated SARS-CoV-2 vaccine formulated with a toll-like receptor 7/8 agonist molecule adsorbed to alum (Algel-IMDG), administered in two doses 4 weeks apart, showed that mean SARS-CoV-2 neutralizing antibody titers (expressed as MNT50) were at a level of 130.3 on day 56 after vaccination and were significantly higher in vaccine recipients who were seropositive for SARS-CoV-2 IgG at baseline than in those who were seronegative (194.3 vs. 118.0). IgG titers for the assayed epitopes (S1 protein, RBD, and N protein) were detected on day 56 (9742, 4124 and 4161 AU/mL, respectively). Neutralizing antibodies to live SARS-CoV-2 declined to below the seropositive cut-off at 6 months after the second dose of vaccine [112].

Seropositivity 28 days after a third dose given at 8 months after primary vaccination was 98–100% regardless of age group. By contrast, a third dose that was given 2 months after the second dose induced much lower neutralizing antibody titers. The reactogenicity of the third dose was indistinguishable from the reactogenicity of the previous two doses, regardless of age group [113].

In another study, the authors found that the neutralization of Omicron was undetectable in participants who had received a two-dose regimen of inactivated vaccine (CoronaVac). The mRNA vaccine booster shot (BNT162b2) resulted in a 1.4-fold increase in neutralization activity against the Omicron variant when compared with the two-dose mRNA vaccine schedule. After this heterologous vaccination (CoronaVac plus BNT162b2), increased virus-specific antibody levels and neutralization activity against the ancestral virus and the Delta variant were observed, resembling the titers obtained after two doses of mRNA vaccines. Despite this increase, neutralizing antibody titers were reduced for Omicron when compared with the ancestral variant (by 7.1-fold) and the Delta variant (3.6-fold). The study confirmed that the Omicron variant is associated with immune escape from vaccines or infection-induced immunity [114].

### 4.4. Recombinant Protein Vaccines

The latest EUL-listed vaccines are recombinant SARS-CoV-2 S-protein Matrix-M1 adjuvanted formulations (NVX-CoV2373—Nuvaxovid, Novavax or Covovax, Serum Institute of India). Matrix-M adjuvant injection induces a local transient pro-inflammatory response with the recruitment, activation, and maturation of immune cells, specifically stimulating DC uptake and processing of co-delivered antigens with their enhanced presentation [115].

21 days after the first dose of NVX-CoV2373, anti-spike IgG was observed for all adjuvanted regimens, and geometric mean fold rises (GMFRs) exceeded those induced without adjuvant by a factor of at least 10. Seven days after the second vaccination, mean titers further increased by a factor of eight when compared to the responses that were seen with the first vaccination, and the titer more than doubled within 14 days, yet again achieving GMFRs 100 times greater than those observed with rSARS-CoV-2 alone. While a single vaccination with adjuvant achieved antibody titers similar to asymptomatic COVID-19 infection, a second vaccination achieved levels six times higher than those of symptomatic COVID-19 outpatients and similar to the levels of patients hospitalized with COVID-19. Neutralizing antibodies had patterns of response similar to those of anti-spike antibodies. The assessment of cellular responses showed antigen-specific polyfunctional CD4+ T cell responses with IFN-γ, IL-2, and TNF-α production on spike protein stimulation. A robust bias toward the Th1 phenotype was noted, while Th2 responses (as measured by IL-5 and IL-13 cytokines) were minimal [116].

A study by Formica et al. [117] compared the safety and immunogenicity of the NVX-CoV2373 vaccine among younger and older patients (aged 18–59 and 60–84). The authors showed increased mean titers for the IgG anti-spike protein after a two-dose regimen in younger individuals (65,019 EU/mL) and lower mean titers in older recipients (28,137 EU/mL). Virus-neutralizing antibody titers were also higher in younger participants (2201 vs. 981) with seroconversion rates of 100% in both age groups. Neutralizing antibody responses exceeded those seen in a panel of convalescent sera for both age groups. 

### 4.5. Persistance of Immunity after Anti SARS-CoV-2 Vaccination

Anti-SARS-CoV-2 vaccination results in both cellular and humoral responses, achieving early control over the spread of the virus. However, longitudinal studies showed the the level of immunization diminishes with time. In a study of 2653 BNT162b2-vaccinated subjects, antibody titers decreased by up to 38% each subsequent month, and at six months after vaccination 16.1% subjects presented antibody levels below the seropositivity threshold [118]. The study involving 344 medical personnel vaccinated with two doses BNT162b2 showed that antibody titers fell by almost 90% within 7–9 months after vaccination [119]. 

Recent papers analyzed the longevity of immunity following COVID-19 vaccination for available vaccines. Observational studies, analyzed in a recent review, stratified by the time since vaccination, identified a decreasing effectiveness at 4–6 months (42–57%) for mRNA vaccines and 47.3% for AZD1222 against Delta infection [120]. Most studies reported that the mRNA vaccines induced neutralizing antibodies, which persisted between 3 to 6 months after the second dose. However, in the context of populational immunity, we have to consider that it is difficult to assess whether the reduction in vaccine effectiveness against new SARS-CoV-2 variants of concern results from waning immunity over time or/and variants escaping immunity.

### 4.6. Effectivenes of Vaccines against Current SARS-CoV-2 Variants of Concern (Delta, Omicron)

As the SARS-CoV-2 is a highly mutating virus, there is a question on the efficacy of available vaccines against novel variants. There is a number of researches studying efficacy against the last two variants of concern—Delta and Omicron. The assessment of particular vaccines’ efficacy is difficult because the new SARS-CoV-2 variants are emerging in the situation when populations are predominantly vaccinated or convalescents [121,122,123,124]. 

Recently published metaanalyses and systematic reviews have analyzed available studies on currently used vaccine regimens. Cortés-Sarabia et al. [125] showed that antibodies developed against the S protein produced by the BNT162b2 vaccine, possessed lower neutralizing activity against the Alpha, Beta, Gamma, and Delta variants of SARS-CoV-2. 

The vaccine efficacy depends not only on the predominant virus variant, but also the timing of vaccination and number of doses. Additionally, a growing number of patients were vaccinated with a heterologous regimen, with two different vaccines used as a primer and a booster [126]. All one- and two-dose regimens failed in effectiveness against Delta and Omicron variants. A booster third dose is required to achieve the vaccine effectiveness agains new variants of concern [127]. 

The summary full vaccination efficacy (VE) against Delta, based on 33 research papers, was shown to be 77.8%. For special populations, the efficacy was 59.7% for the elderly and 88.8% for adolescents. The highest VE was shown for mRNA vaccines (83.4% in 28 studies). In case of vector vacccines, VE was 65% in 12 studies and for inactivated vaccines it was 56.7% in six studies. One study on protein subunit vaccines presented a VE of 78.7%. The highest 95% VE against Delta variant was obtained after booster vaccination. Only three studies analyzed the efficacy of mRNA vaccines against Omicron showing a VE of 55.9% for full vaccination and 80.8% for booster regimens [128]. 

### 4.7. Medications Influencing the Effectivenes of Vaccines against SARS-CoV-2

It is not suprising that immunosuppressive therapy can diminish the effectiveness of anti-SARS-CoV-2 vaccination. In most countries it is recommended that highly immunocompromised patients (treated with anti-CD20 antibodies, mycophenolate mofetil/sodium, or cyclophosphamide) are vaccinated at least 1 month after the therapy. Studies on antibody titers following the first dose of vaccine showed that less than 20% of solid transplant recipients presented detectable antibodies, with the lowest response in those receiving antiproliferative medications. Kidney transplant recipients presented weak anti-SARS-CoV-2 antibody response, with a seroconversion rate of 6.2–10.8% after the first dose [129,130,131].

A recent study on kidney transplant recipients outlined the serological response in 19.5% patients after basic immunizations, with an increase to 29.4, 55.6, and 57.5% after the third, fourth, and fifth vaccination dose, respectively. The cumulative response rate reached 88.7%. The adjusting of immunosuppression by pausing mycophenolate and adding 5 mg prednisolone equivalent before the fourth vaccination increased the serological response rate to 75% in comparison to the no-dose adjustment (52%) or dose reduction (46%). The worst results were noticed for the recipients receiving belatacept-based therapy with a response rate of 12.5% after four dosage vaccination regimen [132]. 

Data on patients with inflammatory bowel disease (IBD) treated with high doses of systemic corticosteroids, infliximab, or infliximab and immunomodulators showed that they present a blunted response to the anti-SARS-CoV-2 vaccination [133]. However, a study on humoral and spike-specific T cell responses in patients with IBD who were on antimetabolite therapy (azathioprine or methotrexate), TNF inhibitors, and/or other biologic treatment (anti-integrin or anti-p40) for up to 6 months after completing two-dose COVID-19 mRNA vaccination showed that a spike-specific T cell response was not only induced in treated patients with IBD at levels similar to those of healthy individuals, but also was sustained at a higher magnitude for up to 6 months after vaccination, particularly in those treated with TNF inhibitor therapy [134]. 

Anti-CD20 therapy depletes B cell populations and thus diminishes the vaccine effectiveness. Patients with rheumatic diseases treated with rituximab presented very low vaccine effectiveness with 30% seronegativity and only a 10% detectable neutralizing response after two doses of mRNA vaccine, and 70% of patients presented a weak T cell response [135]. 

In the study on patients with psoriasis, who were receiving methotrexate or targeted biological monotherapy (TNF-, IL-17- or IL-23 inhibitors), median seroconversion rates were lower in patients receiving immunosuppressants than in controls (60% vs. 100%), with the lowest rate for methothrexate (47%). Neutralizing activity against wild-type SARS-CoV-2 was significantly lower in patients receiving methotrexate, but was preserved in those receiving targeted biologics. Cellular immune responses were not attenuated in patients receiving methotrexate or targeted biologics compared to healthy controls [136]. Another study reported a very low (14.5%) rate of humoral response after the third COVID-19 vaccine dose in patients with autoimmune diseases treated with rituximab who were non-responders to two doses [137]. 

## 5. Immune Response in Special Patient Populations

### 5.1. Elderly Patients

It was assumed that the age of the vaccinated individuals had a significant negative correlation with S-RBD IgG response. In addition to diminished post-vaccine responses, older individuals had a more rapid waning of antibodies after the vaccinations. 

In the real-life study, the authors compared the induction of immune responses in two age groups—individuals aged above 80 were compared to ones below the age of 60 after the first and second vaccination. A total of 176 volunteers were analyzed for vaccine-induced SARS-CoV-2 spike specific IgG titers and SARS-CoV-2 neutralizing antibodies. After the first vaccination, the mean titer for the younger group was 313.3 BAU/mL, as compared to 41.2 BAU/mL in the older one. In this group, 65.9% showed titers below the cut-off. After the second dose, a neutralization titer was attained by 97.8% of members of the younger age group and 68.7% of members of the older age group, respectively [138]. 

In a cohort of 24977 adults who received either the ChAdOx1 or the BNT162b2 SARS-CoV-2 vaccine, 82.1% had a positive post-vaccine anti-spike IgG result with seropositivity dropping faster for those aged >75 years. For both vaccines, low-response participants were older, had mean IgG levels below the positivity threshold throughout, and their response was delayed [139]. In an open-label clinical trial of mRNA-1273, participants ≥56 years were stratified into two subgroups: those between the ages of 56 and 70 years and those who were 71 years of age. The administrated vaccine doses were 25 μg or 100 μg in both groups. The 100-μg dose induced higher binding and neutralizing antibody titers than the 25-μg dose. Among the participants who received the 100-μg dose, the authors did not observe systematic differences in the reactogenicity profile between this older cohort and participants aged 18–55 [140].

Interestingly, in another clinical study after booster vaccination, a specific antibody response to the SARS-CoV-2 spike glycoprotein and RBD was found to be unrelated to dose regimen or age group across all age groups, including adults aged 70 and older. Similar patterns were observed with respect to neutralizing antibody responses [107]. 

### 5.2. Children and Adolescents

It was found that two doses of the inactivated vaccine were safe and well-tolerated among children and adolescents aged 3–17 years old. The seroconversion rates for neutralizing antibodies on day 28 after the second dose were 100% at doses of 1.5 μg and 3.0 μg. The neutralizing antibody titers induced by a 1.5 μg dose vaccine were lower than those of the 3.0 μg dose [141]. A randomized, placebo-controlled, observer-blinded, phase III trial assessing a two-dose regimen of 30 μg of an mRNA vaccine revealed no significant differences in the geometric mean 50% neutralizing titer between 12-to-15-year-old and 16-to-25-year-old participants. Among the 1983 participants in the 12-to-15-year-old group, vaccine efficacy against SARS-CoV-2 was 100% [142]. 

In another study in the United States, Spain, Finland, and Poland, 1517 children aged 5–11 were randomly assigned to receive an mRNA vaccine. The serum-neutralizing geometric mean titers at 7 days after the second dose amounted to 4163 for the 10 μg dose and 4583 for the 20 μg dose. Based on these safety and immunogenicity findings, the 10 μg dose level was selected for further assessment in 5-to-11-year-olds. In phases II to III, the serum-neutralizing geometric mean titers 1 month after the second dose amounted to 1198 in 5-to-11-year-olds and 11 among placebo recipients, while the observed vaccine efficacy was 90.7% among mRNA vaccine recipients [143]. In a randomized, double-blind, controlled study, in phases I and II, healthy participants were stratified according to age (3–5 years, 6–12 years, or 13–17 years) and dose (2, 4 or 8 μg) group. Humoral responses against SARS-CoV-2 reached 100% on day 56 for all three dose levels of all three age cohorts [144].

### 5.3. Pregnant and Lactating Women

Pregnancy and lactation represent distinct immunological states that have been previously associated with reduced immunogenicity. Due to the analysis including pregnant, lactating, and non-pregnant women aged 18–45 who were vaccinated, the median binding antibody titer among pregnant women was 27,601 following vaccination as compared to 37,839 in non-pregnant women. The median cellular responses in vaccinated pregnant women were two-fold lower than in non-pregnant ones. The detection of binding and neutralizing antibodies in infant cord blood suggested the efficient transplacental transfer of maternal antibodies [145]. 

The study on the impact of physiological changes induced by lactation on SARS-CoV-2 vaccination responses revealed that the peak neutralization responses and durability at 4 months against SARS-CoV-2, as well as Beta and Gamma VOCs, was two-fold higher in post-partum women when compared with non-post-partum women following mRNA vaccination. The authors also observed a significantly higher affinity of antibodies to the RBD in post-partum women after the second dose [146]. 

A system serology analysis of the humoral immune response demonstrated similar antibody titers and delayed kinetics of FcR-binding and antibody effector functions in both pregnant and lactating women when compared to non-pregnant women. Additionally, differences were found in the overall antibody profile between women receiving mRNA-1273 and BNT162b2. Women receiving BNT162b2 generated a broader coordinated immune response, including IgG2 and IgM responses and the exclusion of monocyte phagocytosis [147]. The result of a study in 122 pregnant women with cord blood available at the time of birth indicated that maternal IgG levels were significantly higher, week by week, starting 2 weeks after the first vaccine dose, and were linearly associated with cord blood IgG levels. The placental transfer ratio, correlated with the number of weeks elapsed since maternal vaccine dose two, suggests that the timing between vaccination and birth may be an important factor to consider in vaccination strategies for pregnant women [148]. The study included 32 breastfeeding women who were vaccinated during the lactation period. The immune response to the vaccination against SARS-CoV-2 (IgG, IgA) was strongest 7 days after the second dose of the vaccine and milk IgG levels highly correlated to serum IgG levels. The study showed that lactating mothers breastfeeding their children after vaccination against SARS-CoV-2 may transfer antibodies to their infants [19].

### 5.4. Organ Transplant Recipients

Solid organ transplant recipients are a group of immunocompromised patients receiving long-lasting immunosuppression treatment, resulting in a weakened T cell-mediated immunity, making them a vulnerable population with a high risk of severe infections and an increased infection-related mortality rate ranging from 17.9% to as high as 60% short-term fatality rate in elderly KTRs (kidney transplant recipients). Most studies reported a mortality rate of around 20% among hospitalized recipients [44]. It was reported that convalescents after kidney transplantation presented similar serum levels of anti-SARS-CoV-2 IgG and IgA to patients on hemodialysis, but significantly weaker T cell-mediated response [20]. Based on data from other vaccinations, the immune response of solid organ recipients to vaccination may be reduced when compared to healthy vaccine recipients.

In the group of 136 kidney transplant recipients, only 37.5% had a positive antibody response to spike protein when compared to the healthy control group [149]. In infection-naïve groups of kidney transplant recipients, an S-specific immune response with a median antibody IgG titer of 82 AU/mL was seen only in 47.6%. Previous exposure to SARS-CoV-2 infection significantly improved the response to vaccination [150]. Another study reported that the immunization rate among kidney transplant recipients who received two doses of the mRNA-1273 SARS-CoV-2 vaccine was 48% [151]. Bertrand et al. [152] demonstrated that one month after the second dose of mRNA vaccine, the antibody response was scarcely induced in immunocompromised KTRs and was detected in only 17.8% of recipients, as compared to 88.9% in the case of hemodialysis patients. Median antibody titers in responders were 671 AU/mL in kidney transplant recipients and 1052 AU/mL in patients receiving hemodialysis. One month after the second dose, a specific T cell response was detected in 57.8% of recipients and all hemodialysis patients. 

In a six-month observation, SARS-CoV-2-specific anti-spike antibodies were detectable in 12.5% of kidney transplant recipients after the first vaccination, and in 39.5% after the second dose. A SARS-CoV-2 antigen-specific cellular response was detected in 33.3% of recipients following the first dose, and in 60% following the second dose [153]. Only 47.5% of liver transplant recipients had a positive serological response and the mean SARS-CoV-2 S1/S2 IgG titer was significantly lower when compared to the control group [154]. The study in cardiothoracic transplant recipients demonstrated a lack of immunogenicity of the completed prime-boost vaccination with the mRNA SARS-CoV-2 vaccine even 3 weeks after the second dose [25]. 

Due to the poor vaccination response in all solid organ transplant recipients, a third vaccination dose was recommended. In the randomized trial of a third dose of mRNA-1273 vaccine in organ transplant recipients, a third dose of mRNA vaccine had substantially higher immunogenicity than the placebo. The median per cent virus neutralization following the third dose was 71% in the mRNA-1273 group and 13% in the placebo group, and the percentage of patients above the 30% threshold for neutralizing antibody positivity was 60 and 25%, respectively [155]. While most studies demonstrated a substantial benefit of the third dose of SARS-CoV-2 mRNA vaccine in solid organ transplant recipients, a significant proportion of solid organ transplant recipients remain seronegative even after the third vaccine dose [156]. 

### 5.5. Patients with Systemic Rheumatic Diseases

The data concerning the generation of protective antibody titers in patients receiving immunosuppressive therapies for chronic inflammatory diseases are lacking. Two to six weeks after the second mRNA vaccine dose the level of serum IgG neutralizing antibody against SARS-CoV-2 trimeric spike S1/S2 glycoproteins was significantly reduced in patients with autoimmune inflammatory rheumatic diseases when compared with the general population. In patients with systemic lupus erythematosus, axial spondyloarthritis, psoriatic arthritis, and large vessel vasculitis, the seropositive rate was above 90%. The seropositivity rate of patients with rheumatoid arthritis was 82.1% while in participants with idiopathic inflammatory myositis it was <40%. In patients treated with glucocorticoids, the humoral response was detected in only 66% of individuals. Immunogenicity was also impaired in participants treated with abatacept, mycophenolate mofetil, and rituximab, and was mildly impaired in those receiving methotrexate [157]. 

Anti-SARS-CoV-2 spike IgG+ binding neutralizing antibody titers and circulating S-specific plasmablasts to determine the humoral response after mRNA vaccination were assessed in a cohort of 133 participants with chronic inflammatory diseases. The authors found that 88.7% of patients with chronic inflammatory diseases had antibody responses after two doses of vaccine; however, in many cases, antibody levels were lower than in immunocompetent controls. In patients treated with prednisone, 65% were anti–SARS-CoV-2 spike IgG seropositive when compared with 92% among participants who were not using prednisone. No difference in immunogenicity was found in patients receiving antimetabolites [158]. 

In a prospective multicenter study in patients with systemic autoimmune and autoinflammatory rheumatic disease, the humoral response rates were 84.5% in patients receiving immunomodulatory therapy and 97.56% in non-immunosuppressed patients. It was shown that extended treatment modifications regarding mycophenolate mofetil, rituximab and methotrexate improve the immunogenicity of mRNA vaccines without significantly affecting the disease activity status [159]. 

### 5.6. Patients with Hematological Malignancies

In a multicenter prospective observational study assessing the anti-SARS-CoV-2 mRNA vaccine among patients with lymphoid malignancies receiving systemic anti-lymphoma therapy, 72% of patients after one dose and 61% of patients after two doses of the vaccine had no detectable anti-S IgG antibodies. Among the participants with lymphoma who were not undergoing treatment, all patients with Hodgkin lymphoma and 81% of patients with aggressive B cell non-Hodgkin lymphoma developed robust (81–100%) serological responses [160]. Only 47% of chronic lymphocytic leukaemia patients developed a positive humoral response, followed by 60% of indolent non-Hodgkin lymphoma, 71% of aggressive non-Hodgkin lymphoma, 76% of multiple myeloma, 80% of acute leukaemia and 84% of myeloproliferative neoplasm patients, as well as 91% of chronic myeloid leukaemia patients, and 94% of Hodgkin lymphoma and myelodysplastic syndrome patients. 

The type of treatment at vaccination significantly affected the rate of seropositivity. The lowest rate of seropositivity (0%) was exhibited by patients receiving a single-agent anti-CD20 therapy; patients taking BCL2 inhibitors had a rate of 25%, patients receiving chemo-immunotherapy a rate of 29%, whereas those receiving BTK and JAK2 inhibitor treatment had rates of 40 and 42%, respectively. Patients who underwent auto-stem cell transplantation had the same rate of seropositivity as those who did not [161]. A Lithuanian prospective cohort study on the serological response to one and two mRNA vaccine doses in patients with hematological malignancies showed heterogeneous and markedly blunted serological responses to two doses of mRNA vaccine in patients with hematological malignancies, regardless of age or treatment. Nevertheless, after two doses, the median anti-S1 IgG antibody concentration in the group that had received Bruton tyrosine kinase inhibitor treatment less than 12 months before vaccination was 0 AU/mL; for venetoclax, it was 4 AU/mL, for ruxolitinib it was 10 AU/mL and for anti-CD20 antibodies it was 17 AU/mL, compared to 5761 AU/mL in untreated patients [162]. 

Regarding the efficacy of the third dose of the anti-SARS-CoV-2 vaccine, the results reported were similar to those in organ transplant recipients. A heterologous vaccination regimen combining mRNA and a vectored vaccine led to a serological response in 31% of hemato-oncological patients who did not respond after a previous double dose of mRNA vaccine [163]. 

Frequent and high levels of IgG (S-RBD) titers were observed in the first evaluation of immunogenicity in allogeneic hematopoietic stem-cell transplant recipients after two vaccine doses. Low humoral responses were detected in patients with a lymphocyte count below 1 G/L in peripheral blood, who were treated with systemic immunosuppressive treatments within 3 months of vaccination. The administration of the third dose of the mRNA vaccine improved the immunogenicity of the vaccine in this group of patients [162,164]. In a multicenter prospective observational study, the anti-S IgG detection rate was 78% among 311 allogeneic and 85% among 86 autologous hematopoietic stem cell transplant recipients. Lower probabilities of detectable antibodies were observed in patients who had lymphopenia < 1 × 10^9^/mL, active graft-versus-host disease or were vaccinated less than 1 year after transplantation [165]. 

### 5.7. Patients with Solid Malignancies

Besides seroconversion, T cell responses in this immunosuppressed population have emerged as an important tool to address the immunogenicity against SARS-CoV-2. In a prospective, longitudinal observational study of patients with cancer, a single dose of 30 μg mRNA vaccine failed to induce seroconversion in most patients. Anti-SARS-CoV-2 IgG response was detected in 38% and T cell vaccine response in 71% of patients with solid cancer compared to 94 and 82% in healthy controls, respectively. The main difference between cancer patients and healthy controls was a failure to produce a response rather than the magnitude of the response. A total of 41% of solid cancer patients received anticancer treatment within 15 days preceding day 1 vaccination [166]. 

After the second dose of mRNA vaccine in cancer patients, the anti-S CD4+ T cell response rate was 57.9%; for anti-S CD8+ T, it amounted to 55.6%, while the rate of specific anti-S IgG was 95.8%. The immunogenicity in the healthy control group was significantly higher and amounted to 91.7, 94.4 and 100%, respectively. The magnitude of a response measured by the percentage of IFN-g producing CD4+ and CD8+ T cells was also significantly lower in patients treated with CDK4/6 inhibitors than those treated with chemotherapy or immunotherapy [167]. 

A significant difference in serological anti-S IgG responses was noted between the various anti-cancer treatment modalities. Lower levels of antibody titer were observed in patients treated with monoclonal antibody therapy and cytotoxic chemotherapy when compared to the group of patients receiving endocrine therapy, with seroconversion rates of 98 to 100% and a median antibody titer of >2500 U/mL [168]. In an observational study including 257 patients, IgG antibody response against subunits S1 and S2 of the SARS-CoV-2 spike protein was detected in 82.7% of non-metastatic cancer patients and 72.3% of metastatic participants. In patients with gastrointestinal cancer, only 67.1% developed a positive humoral response, followed by 67.7% with lung cancer, 80% with gynaecological and 84.2% with breast cancer [169].

The particular studies concerning effectiveness of different vaccines againd COVID-19 in special patients populations are summarized in Table 2.

## 6. Conclusions

Numerous studies have focused so far on the role of the immune system in the development of COVID-19 symptomatology and immune response to vaccination. 

In general, the immune responses generated after SARS-CoV-2 infection or vaccination are similar, however they differ in the details. Below, we briefly summarize the findings published in the manuscript cited papers regarding both types of response.

After the SARS-CoV-2 infection induces innate immunity activation, the cellular response starts from NK cells, through cytokine production (mainly IL-6) and the direct lysis of infected cells, followed by CD8+ T cells’ destruction of infected cells, and CD4+ T cells stimulating B cells to produce antibodies. Persistant infection leads to further T cell exhaustion with lymphopenia with a high CD8+/CD4+ ratio as a major hallmark of progression to severe COVID-19.

The take-up of the mRNA vaccine by a cell results in an expression of the SARS-CoV-2 antigen that can be processed for presentation via class I and II MHC from the transfected cells and professional antigen-presenting cells, respectively. As a result, Th1-cell-skewed immune response can be generated with antigen-specific CD4+ T helper and CD8+ cytotoxic T cells, along with neutralizing antibody responses from B cells. The vaccination-induced activation of the innate immune cells is also required to activate the lymphocytes to obtain both T cell response and B cell-related virus-neutralizing activity.

The adenovirus-vectored SARS-CoV-2 vaccines mimic the features of natural viral infection. As a result of adenovirus vector infection, an infected cell expresses the vector-encoded S protein of SARS-CoV-2 on the cell membrane, leading to Th1-cell-skewed response, also followed by a neutralizing antibody response from B cells.

Matrix-M adjuvant vaccination also leads to robust bias toward the Th1 phenotype, with minimal Th2 responses and neutralizing antibody production.

A number of structural and non-structural SARS-CoV-2 proteins can elicit the host immune response, however antibodies directed against the SARS-CoV-2 spike RBD are considered to be responsible for neutralization of the virus. All EMA and/or FDA authorized anti-SARS-CoV-2 vaccines are based on the spike sequence. As a result, the post-vaccination immune response in directed solely against spike RBD. In the case of SARS-CoV-2 convalescents, there is a broader spectrum of antibodies, also including those directed against nucleocapsid protein.

The WHO EUL portfolio includes also inactivated virus vaccines (authorized by other than EMA or FDA agencies) that are similar to the live virus infection, however, they offer a limited duration of the presented antigens and lack dead infected cell fragments for MHC I presentation of the viral antigens. The initial results of clinical trials of inactivated virus vaccines have suggested a Th2-skewed T cell response to that type of vaccination. The difference between inactivated whole-virion formulation and mRNA or adenoviral-vectored vaccines is that the viral antigens (S, M and N structural proteins) from inactivated virions can be directly presented to DCs after their uptake through the lysosomal pathway. An immune reaction to inactivated-virus vaccination mimics the natural SARS-CoV-2 infection with a broader spectrum of generated specificity, beyond spike-only directed responses.

While many aspects of immunity in COVID-19 have been already explained, there is still a gap between the expectations of clinicians and the available explanations, particularly in the context of SARS-CoV-2 evolution and novel emerging variants.

The strength and durability of immunity acquired after infection or vaccination against SARS-CoV-2 are generally similar with slightly higher post-vaccination antibody titers and a somewhat longer duration of post-infectious response. However the published data are inconsistent. The only well-proven fact is the much stronger post-vaccination immune response achieved by convalescent patients. Some authors described sustained cellular immunity despite the antibody decline, suggesting other immune-related components contributing to protective immunity. 

In conclusion, despite the enormous efforts of scientists worldwide, the durability of immunity against SARS-CoV-2, especially in the context of potentially emerging novel variants of concern, remains unclear and the only proven strategy to reduce mortality and morbidity is social distancing, as well as generalized vaccination.

## Figures and Tables

**Table 1:** Vaccines against SARS-CoV-2 emergency use listed by the World Health Organization (WHO).

Vaccine	National Regulatory Agency (Date of EUA *)
Nucleoside Modified Vaccines	
Comirnaty^®^ COVID-19 mRNA Vaccine (Pfizer/BioNTech, Mainz, Germany)	European Medicines Agency (31 December 2020) Food and Drug Administration (16 July 2021)
Spikevax COVID-19 mRNA Vaccine (Moderna, Madrid, Spain)	European Medicines Agency (30 April 2021) Ministry of Food and Drug Safety (MFDS), Republic of Korea (23 December 2021) Food and Drug Administration (6 August 2021)
Vectored vaccines	
Vaxzevria COVID-19 Vaccine, ChAdOx1-S (AstraZeneca, Cambridge, Great Britain)	Ministry of Food and Drug Safety (MFDS), Republic of Korea (15 February 2021) European Medicines Agency (15 April 2021) Ministry of Health, Labour and Welfare, Japan (9 July 2021) Therapeutic Goods Administration, Australia (9 July 2021) Health Canada (21 August 2021) COFEPRIS (DP) and ANMAT (DS), Mexico and Argentina (23 December 2021)
Covishield™ COVID-19 Vaccine, ChAdOx1-S (Serum Institute of India Pvt. Ltd., Pune India)	Central Drugs Standard Control Organization, India (15 February 2021)
Jcovden COVID-19 Vaccine, Ad26.COV2-S (Janssen Pharmaceutica, Beerse, Belgium)	European Medicines Agency (12 March 2021)
Convidecia COVID-19 Vaccine, Ad5.CoV2-S (CanSino Biologics, Tianjin, China)	National Medicinal Products Administration (NMPA), China (19 May 2022)
Inactivated vaccines	
Inactivated COVID-19 Vaccine (Vero Cell) (Beijing Institute of Biological Products Co., Ltd., Beijing, China)	National Medicinal Products Administration (NMPA), China (7 May 2021)
CoronaVac COVID-19 Vaccine (Vero Cell) (Sinovac, Beijing, China)	National Medicinal Products Administration (NMPA), China (1 June 2021)
Covaxin^®^ COVID-19 vaccine, Whole Virion Inactivated Corona Virus vaccine (Bharat Biotech, Hyderabad, India)	Central Drugs Standard Control Organization, India (3 November 2021, currently suspended)
Protein vaccines (Matrix-M1 adjuvanted)	
Covovax™ COVID-19 vaccine, SARS-CoV-2 rS (Serum Institute of India PVT. Ltd., Pune India)	Central Drugs Standard Control Organization, India (17 December 2021)
Nuvaxovid™ COVID-19 vaccine, SARS-CoV-2 rS (Novavax, Bohumil, Czech Republic)	European Medicines Agency (20 December 2021)

* EUA—Emergency Use Authorization.

**Table 2:** The selected studies concerning effectiveness of European Medicines Agency and/or Food and Drug Administration authorized vaccines against COVID-19 in special patients populations.

Vaccine	Special Populations
Comirnaty^®^ COVID-19 mRNA Vaccine	Elderly patients [138,139] Children & adolescents [142,143] Pregnant & lactating women [19,145,146,147,148] Solid organ transplant recipients [25,149,150,152,153,154,156] Systemic rheumatic diseases [157,158,159] Hematological malignancies [160,161,162,163,164,165] Solid malignancies [166,168,169]
Spikevax COVID-19 mRNA Vaccine	Elderly patients [140] Pregnant & lactating women [145,146,147,148] Solid organ transplant recipients [151,155,156] Systemic rheumatic diseases [158,159] Hematological malignancies [165] Solid malignancies [167,168,169]
Vaxzevria COVID-19 Vaccine (ChAdOx1-S)	Elderly patients [107,139] Hematological malignancies [160]
COVID-19 Vaccine (Ad26.COV2-S)	Hematological malignancies [163]
Nuvaxovid™ COVID-19 vaccine (SARS-CoV-2 rS)	Elderly patients [117]

## Data Availability

Not applicable.
